# Prevalence, predictors, and prognosis of symptomatic intracranial stenosis in patients with transient ischaemic attack or minor stroke: a population-based cohort study

**DOI:** 10.1016/S1474-4422(20)30079-X

**Published:** 2020-05

**Authors:** Robert Hurford, Frank J Wolters, Linxin Li, Kui Kai Lau, Wilhelm Küker, Peter M Rothwell, Oxford Vascular Study Phenotyped Cohort

**Affiliations:** a Wolfson Centre for the Prevention of Stroke and Dementia, Nuffield Department of Clinical Neurosciences, University of Oxford, Oxford, UK

## Abstract

**Background:**

Symptomatic intracranial stenosis was perceived to convey a high risk of recurrent stroke, but two previous trials (SAMMPRIS and VISSIT) did not show superiority of intracranial stenosis stenting over intensive medical management alone. These findings were partly due to a lower than expected risk of recurrent stroke without stenting, possibly reflecting the young age of recruits (median age <60 years), and raise questions about generalisability to routine clinical practice. We therefore studied the age-specific prevalence, predictors, and prognosis of symptomatic intracranial stenosis in a population-based cohort of patients with transient ischaemic attack and minor stroke on intensive medical management.

**Methods:**

The Oxford Vascular Study (OXVASC) is a prospective incidence cohort study of all vascular events in a population of 92 728 people residing in Oxfordshire, UK. All patients, irrespective of age, with transient ischaemic attack and minor ischaemic stroke occurring between March 1, 2011, and March 1, 2018 (follow-up to Sept 28, 2018), were ascertained with multiple methods, including assessment in a dedicated daily emergency clinic and daily review of all hospital admissions. Imaging was by MR angiography of the intracranial and cervicocranial arteries, by CT angiography if MR angiography was contraindicated, and by transcranial Doppler and carotid ultrasound if CT angiography was contraindicated. All patients received intensive medical treatment without stenting, and those with intracranial vascular imaging were analysed in our study, which assessed the age-specific prevalence of 50–99% intracranial stenosis and the associated stroke risk of 50–99% and 70–99% stenosis (adjusted for age and vascular risk factors) during follow-up to Sept 28, 2018.

**Findings:**

Of 1368 eligible patients with intracranial vascular imaging, 241 (17·6%) had 385 50–99% symptomatic or asymptomatic intracranial stenosis. The prevalence of symptomatic 50–99% intracranial stenosis increased from 29 (4·9%) of 596 at younger than 70 years to 10 (19·6%) of 51 at 90 years or older (p_trend_<0·0001). Of 94 patients with 50–99% symptomatic intracranial stenosis, 14 (14·9%) had recurrent strokes (12 ischaemic and two haemorrhagic) during a median follow-up of 2·8 years (IQR 1·5–4·6). Although symptomatic intracranial stenosis conveyed an increased risk of ischaemic stroke compared with no intracranial stenosis (adjusted hazard ratio 1·43, 95% CI 1·04–1·96), the risk of same-territory ischaemic stroke in patients with 70–99% symptomatic intracranial stenosis tended to be less than those reported in the non-stenting groups of the previous trials (1-year risk 5·6% [95% CI 0·0–13·0] *vs* 9·4% [3·1–20·7] in VISSIT; 2-year risk 5·6% [0·0–13·0] *vs* 14·1% [10·1–19·4] in SAMMPRIS).

**Interpretation:**

The prevalence of 50–99% symptomatic intracranial stenosis increases steeply with age in predominantly Caucasian patients with transient ischaemic attack and minor ischaemic stroke. However, the risk of recurrent stroke on intensive medical treatment of symptomatic intracranial stenosis is consistent with the two previous randomised controlled trials in younger cohorts, supporting the generalisability of the trial results to routine practice.

**Funding:**

Wellcome Trust, Wolfson Foundation, British Heart Foundation, National Institute for Health Research, National Institute for Health Research Oxford Biomedical Research Centre, Association of British Neurologists.

## Introduction

Intracranial atherosclerotic stenosis of the major cerebral arteries is a common cause of ischaemic stroke.^1^, ^2^ Intracranial stenosis is particularly prevalent in Asians,^3^, ^4^, ^5^, ^6^ but is under-investigated in population-based studies of non-Asians (appendix pp 1–3). Although patients with intracranial stenosis have long been considered to be at high risk of recurrent stroke (appendix pp 4–6), leading to the development and wide use of percutaneous stenting in some countries, two randomised controlled trials did not show provide evidence of benefit for percutaneous stenting over intensive medical management alone in patients with recently symptomatic intracranial stenosis: Stenting Versus Aggressive Medical Management Therapy for Intracranial Arterial Stenosis (SAMMPRIS)^7^ and Vitesse Intracranial Stent Study for Ischemic Stroke Therapy (VISSIT).^8^ Both trials were stopped early partly owing to lower than expected recurrent stroke rates in the medical management groups; SAMMPRIS reported a 2-year risk of death or stroke in the intracranial stenosis territory of 14·1% (95% CI 10·1–19·4), and VISSIT reported a 1-year risk of ischaemic stroke or transient ischaemic attack in the intracranial stenosis territory of 15·1% (6·7–27·6).^8^, ^9^

**Research in context**

**Evidence before this study**

We did two systematic reviews, one on the prevalence and one on the prognosis of intracranial stenosis in population and hospital-based cohorts of patients with transient ischaemic attack and ischaemic stroke. We searched Embase and Medline databases for articles published in English from database inception to Nov 1, 2019 with the search terms “[prevalence] OR [prognosis] AND [intracranial stenosis]” (appendix pp 16, 17). Studies were chosen if they had been done in mostly Caucasian patients with ischaemic attack or stroke (or in European centres if ethnicity not specified) receiving medical treatment only. 50 studies, including 25 prognostic studies, were identified.

Multiple imaging methods for screening for intracranial stenosis were used in 21 studies, 15 of which included transcranial Doppler, transcranial Doppler only was used in 11 studies, CT angiography or MR angiography in seven, and catheter angiography in 11. Intracranial stenosis definition included a 50% reduction of the luminal diameter (usually by the Comparison of Warfarin and Aspirin for Symptomatic Intracranial Arterial Stenosis method) in 28 studies, by transcranial Doppler flow criteria in 11 studies, other percentage reductions in six studies, any reduction in four studies, and was not described in one study. The mean age of participants in all studies was 64 years (SD 6·3). The mean pooled prevalence of any intracranial stenosis was 16·5% (11·7) and of symptomatic intracranial stenosis 10·1% (9·9). However, substantial heterogeneity exists in the reported risk estimates following symptomatic intracranial stenosis (ranging from 4·6% to 45·7%) due to a mixture definitions, case ascertainment, and length of follow-up.

**Added value of this study**

We did a large, prospective population-based longitudinal cohort study of all acute cerebrovascular events, irrespective of age, with near-complete ascertainment and a high rate of intracranial vascular imaging. The prevalence of intracranial stenoses in elderly patients with transient ischaemic attack or minor ischaemic stroke was high in Caucasian patients, but the absolute risk of recurrent ischaemic events was low on intensive medical management. Our findings also validate the risk estimates from two previous randomised controlled trials (SAMMPRIS and VISSIT) in a population-based setting and support the role for intensive medical management over stenting, irrespective of age.

**Implications of all the available evidence**

Estimates of stroke risk distal to symptomatic intracranial stenosis from previous studies are heterogeneous, but imaging methods and outcome definitions differed and intensity of medical treatment and compliance during follow-up were uncertain, such that meta-analysis would be difficult to interpret. Our findings provide risk estimates in an up-to-date population-based setting with standard non-invasive imaging and high rates of guideline-based medical treatment and support the role for medical management of symptomatic intracranial stenosis over routine stenting, irrespective of age.

Although guidelines have reflected the results of SAMMPRIS and VISSIT,^10^, ^11^ there is evidence of ongoing angioplasty or stenting for patients with symptomatic intracranial stenosis in some health-care systems,^12^, ^13^ and the generalisability of the results of the trials has been questioned.^14^, ^15^ Specifically, two non-technical criticisms of the trials have been raised. First, the better than expected prognosis on medical treatment alone in these trials might reflect the exclusion of older patients from both, with a median age at recruitment of younger than 60 years, such that generalisability of the trial results to older patients is uncertain. Second, the particularly intensive medical treatment might have been responsible for the low stroke risks in the medical treatment-only groups compared with previous studies.^16^, ^17^

To understand the external validity of the findings in these two trials of stenting versus intensive medical treatment only for patients with recently symptomatic intracranial stenosis in relation to age and intensive medical treatment, we aimed to determine the age-specific prognosis of symptomatic intracranial stenosis in a population-based cohort of patients with transient ischaemic attack and minor ischaemic stroke (to replicate trial eligibility) recruited irrespective of age and followed-up on intensive medical treatment.

## Methods

### Study design and participants

The Oxford Vascular Study (OXVASC) is a longitudinal population-based prospective cohort study of all incident acute vascular events in a defined population of 92 728, covered by around 100 primary care physicians in nine primary care practices in Oxfordshire, UK.^19^ An estimated 97% of the true study residential population is registered with a primary care practice; most unregistered people are young students. The study area contains a mix of urban and rural populations. The OXVASC population is 94% Caucasian, 3% Asian, 2% Chinese, and 1% Afro-Caribbean.^18^

Written, informed consent or assent from relatives was obtained for all participants for study interview and follow-up, including ongoing review of primary care and hospital records and death certificate data. OXVASC was approved by the Oxfordshire research ethics committee (OREC A: 05/Q1604/70).

### Procedures

We studied consecutive patients referred to OXVASC between March 1, 2011, and March 1, 2018, with transient ischaemic attack or minor ischaemic stroke (defined as National Institute of Health Stroke Scale [NIHSS] score ≤3). Patients who had intracranial vascular imaging were included in our analyses. Only patients with transient ischaemic attack or minor ischaemic stroke were included in this study to reflect the eligibility criteria for SAMMPRIS and VISSIT, both of which excluded major strokes.

Multiple overlapping methods were used to ascertain all individuals with transient ischaemic attack and stroke, approaching 100% of events reaching medical attention. These methods included: a daily, rapid access clinic to which participating general practitioners and the local emergency department refer individuals with suspected transient ischaemic attack or minor ischaemic stroke; daily searches of admissions to the medical, stroke, neurology, and other relevant wards; daily searches of the local emergency department attendance register; daily searches of in-hospital death records through the Bereavement Office; monthly searches of all death certificates and coroner's reports for out-of-hospital deaths; monthly searches of general practitioner diagnostic coding and hospital discharge codes; and monthly searches of all brain and vascular imaging referrals.^20^, ^21^

Demographic data and stroke risk factors were collected in face-to-face interviews by study physicians as soon as possible after referral or hospital admission and cross-referenced with primary care records. Detailed clinical history was recorded for all patients and stroke severity was assessed using the NIHSS. Cause of ischaemic events was classified according to the Trial of Org 10172 in Acute Stroke Treatment criteria.^22^ Transient ischaemic attack was defined according to the National Institute of Neurological Disorders and Stroke criteria, and stroke according to the WHO criteria,^23^ with review of all cases as soon as possible after presentation by the same senior neurologist (PMR) throughout the study.

Patients were followed up face to face at 1, 6, 12, 24, 60, and 120 months by a study nurse or physician to identify any recurrent stroke (supplemented by review of primary care records) and to ensure medication compliance and adequate blood pressure control. Patients who had moved out of the study area (or were unwilling or unable to have face-to-face follow-up) were followed-up by telephone at the same timepoints. All recurrent events that occurred during follow-up would also be identified by the ongoing daily case ascertainment. We recorded all deaths during follow-up with the underlying causes by direct follow-up, through primary care records, and by centralised registration with the Office for National Statistics.

All OXVASC patients received intensive medical management, including dual anti-platelet therapy (aspirin and clopidogrel) for the first month, with aspirin or clopidogrel monotherapy thereafter, high-dose statin therapy, and treatment of hypertension to guideline targets (<130/80 mm Hg). Patients were also provided advice on smoking cessation and diet.

Intracranial vascular imaging has been done routinely in all patients in OXVASC since April 1, 2010. We attempted to obtain imaging for as many patients as possible by using MR angiography as first choice, CT angiography (Aquilion 64, 64-slice scanner; Toshiba, Tokyo, Japan) if MRI was contraindicated (eg, implantable devices or claustrophobia), and transcranial Doppler (Doppler Box; Compumedics DWL, Singen, Germany) and carotid ultrasound if CT angiography was also contraindicated (eg, low estimated glomerular filtration rate).

The MRI scanners and protocols used in OXVASC have been described elsewhere,^25^ but sequences included diffusion-weighted imaging, time-of-flight angiography of the intracranial arteries, and gadolinium contrast-enhanced MR angiography of the intracranial and cervicocranial arteries, including the aortic arch. Patients were scanned at the Acute Vascular Imaging Centre, John Radcliffe Hospital (Oxford), in a Siemens (Erlangen, Germany) Verio 3.0 Tesla scanner; a neurovascular coil was used (contrast-enhanced MR angiography sequence: 15 ml ProHance followed by 40 ml NaCl, flow rate 2 mL/s, repetition time 22 ms, echo time 3·6 ms, flip angle 18°, slice thickness 0·5 mm).

Reconstructed time-of-flight MR angiography sequences were used to assess intracranial stenoses and contrast-enhanced sequences to assess extracranial stenoses. Both sequences were used for assessment of potential artefact. In the case of CT angiography use, unreconstructed CT angiography images were analysed.

Stenoses eligible for inclusion were defined as 50–99% of the luminal diameter, measured using the Comparison of Warfarin and Aspirin for Symptomatic Intracranial Arterial Stenosis (WASID) method^26^ (between the narrowest point and compared with the normal luminal size before the stenosis) or using Stroke Outcomes and Neuroimaging of Intracranial Atherosclerosis criteria with transcranial Doppler.^24^ Symptomatic intracranial stenosis detected by MR angiography or CT angiography were sub-classified into 70–99% stenosis also using the WASID method.^26^ Trained assessors (RH and FJW) independently evaluated the images for vascular stenosis, masked to the clinical details and consultant neuroradiologist report (WK). In situations of disagreement, a third assessor adjudicated (LL). Extracranial arteries assessed included subclavian, common carotid, proximal internal carotid, and vertebral (V1, V2, V3), and intracranial arteries assessed included distal internal carotid, middle cerebral (M1 and M2), anterior cerebral, posterior cerebral (P1 and P2), basilar, posterior communicating, and vertebral (V4). The standard anatomical landmarks used are outlined in the appendix (p 7). Eligible stenoses were classified as symptomatic or asymptomatic in relation to the most recent clinical presentation and results of parenchymal brain imaging.

### Statistical analysis

Baseline characteristics were compared between patients with 50–99% intracranial stenosis versus no intracranial stenosis using χ^2^ or Student's *t* test as appropriate. Baseline characteristics of patients with 50–99% and 70–99% symptomatic intracranial stenosis were also compared with those in the non-stenting groups of the SAMMPRIS and VISSIT trials. Interobserver agreement for 50–99% stenosis was assessed using Cohen's kappa.

We calculated the age-specific prevalence of 50–99% symptomatic and asymptomatic intracranial stenosis and occlusions in 10-year bands in OXVASC and compared them with those for extracranial stenosis. We also determined any other predictors of any 50–99% symptomatic or asymptomatic intracranial stenosis with univariate, age-adjusted, and multivariate regression analyses.

We used Kaplan-Meier survival analysis to determine risk of recurrent ischaemic stroke during follow-up after the index event, stratified by 50–99% symptomatic intracranial stenosis and no intracranial stenosis, including and excluding patients with atrial fibrillation. Analyses were censored at recurrent event, death, or the end of follow-up (Sept 28, 2018). We used Cox regression analysis to compare risks of recurrent ischaemic stroke, ischaemic vascular events (ischaemic stroke, myocardial infarction, or peripheral vascular disease), and death during follow-up in patients with 50–99% symptomatic intracranial stenosis versus no intracranial stenosis and 50–99% asymptomatic intracranial stenosis versus no intracranial stenosis, with adjustment for baseline characteristics that were independent predictors of the presence of intracranial stenosis.

We also used Cox regression analysis to compare risks of outcomes reported in the non-stenting groups of SAMMPRIS and VISSIT trials with comparable outcomes in the OXVASC cohort. Only patients fulfilling the trial inclusion criteria were included for this analysis—ie, patients with 70–99% symptomatic intracranial stenosis, without tandem stenoses, bilateral intracranial vertebral artery stenoses, intracranial arterial occlusion, atrial fibrillation, or cardioembolic cause, and without intracranial stenosis stenting or angioplasty.

All statistical analyses were done with SPSS version 25.0.

### Role of the funding source

The funder of the study had no role in study design, data collection, data analysis, data interpretation, or writing of the report. The corresponding author had full access to all the data in the study and had final responsibility for the decision to submit for publication.

## Results

Of 1579 eligible patients (1000 [63·4%] transient ischaemic attack and 579 [36·7%] minor ischaemic stroke), 1368 (86·6%) underwent intracranial vascular imaging (1034 [65·5%] MR angiography, 253 [16·0%] CT angiography, and 81 [5·2%] transcranial Doppler only), whereas 154 (9·8%) had only carotid bifurcation ultrasound imaging (often due to contraindications to MR and CT angiography) and 57 (3·6%) did not undergo any vascular imaging (appendix p 8). Patients who did not receive intracranial vascular imaging were older with a greater burden of vascular risk factors (appendix p 9).

Of the 1368 patients with intracranial vascular imaging, 385 50–99% intracranial stenoses were identified in 241 (17·6%) patients (table 1). Of 241 patients with any (symptomatic or asymptomatic) 50–99% intracranial stenosis, 188 (78·0%) received MR angiography, 49 (20·3%) CT angiography, and four (1·7%) transcranial Doppler. Prevalence of any 50–99% intracranial stenosis in imaged patients (n=1368) was similar in the intracranial segment of the internal carotid artery (84 [3·4%]), the posterior cerebral artery (93 [3·6%]), and the middle cerebral artery (98 [3·8%]). The basilar artery was the least affected with 13 (1·0%) stenoses (appendix p 10). The vertebral arteries (V1–3; 236 [9·6%]) and proximal internal carotid artery (273 [9·5%]) were the most common sites of extracranial stenosis. There was good agreement of inter-rater reliability for the presence of intracranial (Cohen's kappa 0·82), extracranial (0·79), and no stenosis (0·84; n=50).

**Table 1:** Baseline characteristics

		**Patients with intracranial vascular imaging, N=1368**		**p value**
		Intracranial stenosis, n=241*	No intracranial stenosis, n=1108	
Age, years		76·0 (11·9)	67·7 (13·8)	<0·0001
Sex		..	..	0·40
	Male	127 (52·7%)	558 (50·4%)	..
	Female	114 (47·3%)	550 (69·6%)	..
White ethnicity		229 (95·0%)	1045 (94·3%)	0·56
Hypertension		168 (69·7%)	572 (51·6%)	<0·0001
Diabetes		42 (17·4%)	134 (12·1%)	<0·0001
Hyperlipidaemia		103 (42·7%)	355 (32·0%)	<0·0001
Current smoker		26 (10·8%)	165 (14·9%)	0·090
Atrial fibrillation		52 (21·6%)	145 (13·1%)	0·0010
Any vascular disease†		105 (43·6%)	259 (23·4%)	<0·0001
History of stroke or transient ischaemic attack		58 (24·1%)	143 (12·9%)	<0·0001
Peripheral vascular disease		24 (10·0%)	36 (3·2%)	<0·0001
Ischaemic heart disease		54 (22·4%)	120 (10·8%)	<0·0001
Event type		..	..	0·22
	Transient ischaemic attack	146 (60·6%)	735 (66·3%)	..
	Minor ischaemic stroke	95 (39·4%)	373 (33·7%)	..
TOAST classification		..	..	<0·0001
	Cardioembolic	35 (14·5%)	172 (15·5%)	..
	Atherosclerotic	95 (39·4%)	78 (7·0%)	..
	Undetermined	47 (19·5%)	494 (44·6%)	..
	Lacunar	11 (4·6%)	120 (10·8%)	..
	Multiple, unknown, or other	53 (22·0%)	244 (22·1%)	..
Vascular territory		..	..	0·12
	Carotid	129 (53·5%)	580 (52·4%)	..
	Vertebrobasilar	94 (39·0%)	408 (36·8%)	..
	Uncertain or both	18 (7·5%)	120 (10·8%)	..
Imaging method		..	..	0·039
	MR angiography	188 (78·0%)	829 (74·8%)	..
	CT angiography	49 (20·3%)	202 (18·2%)	..
	Transcranial Doppler	4 (1·7%)	77 (7·0%)	..

TOAST=Trial of Org 10172 in Acute Stroke Treatment.

* Excluding patients with intracranial vessel occlusion (n=19).

† Vascular disease includes ischaemic stroke, transient ischaemic attack, peripheral vascular disease, or ischaemic heart disease.

Patients with intracranial stenoses were older than those without and had a greater burden of hypertension, diabetes, hyperlipidaemia, atrial fibrillation, previous stroke or transient ischaemic attack, peripheral vascular disease, and ischaemic heart disease (all p≤0·0010; table 1). Intracranial stenosis was considered symptomatic in relation to the most recent clinical presentation in 94 (6·9%) patients.

**Figure 1:**
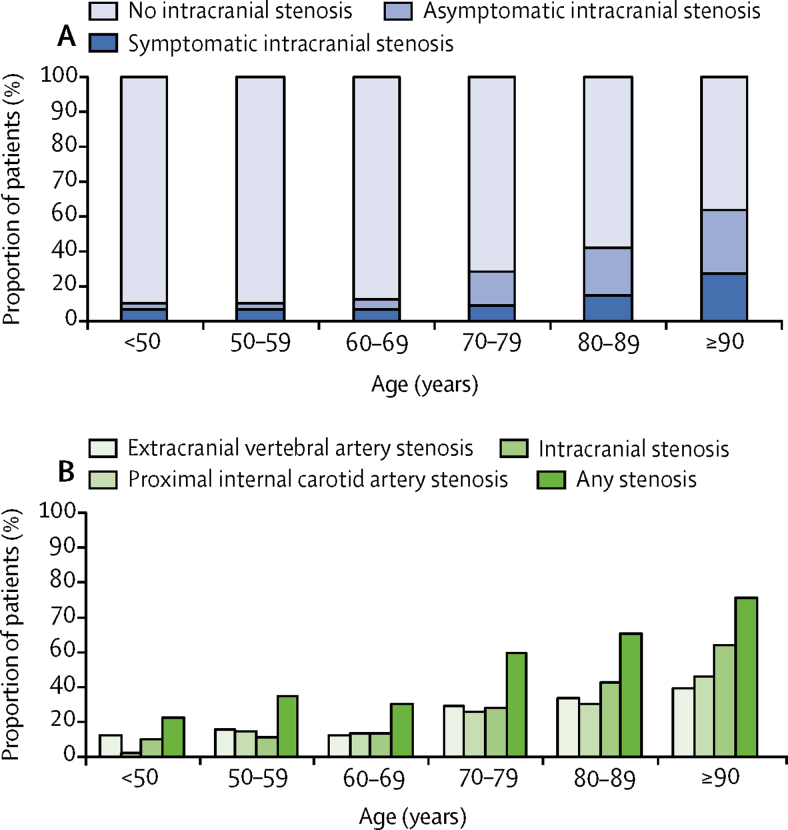
Age-specific prevalence of extracranial and intracranial stenosis (A) 50–99% symptomatic, asymptomatic, and no intracranial stenosis. (B) Proximal extracranial internal carotid artery stenosis, extracranial vertebral artery stenosis, 50–99% intracranial stenosis, and any stenosis (extracranial or intracranial).

The prevalence of any 50–99% intracranial stenosis increased with age from nine (7·0%) of 129 at younger than 50 years to 23 (45·1%) of 51 at 90 years or older (figure 1A), and from six (4·7%) of 129 to 10 (19·6%) of 51 for 50–99% symptomatic intracranial stenosis. At younger than 80 years the prevalence of intracranial stenosis was similar to that of stenosis of the proximal internal carotid artery and to that of extracranial vertebral stenosis (figure 1B), but intracranial stenosis predominated at older ages.

The independent baseline predictors of any 50–99% intracranial stenosis (table 2) were age (odds ratio 1·60, 95% CI 1·39–1·83), history of minor ischaemic stroke or transient ischaemic attack (1·64, 1·13–2.38), peripheral vascular disease (1·94, 1·09–3.45), and presenting minor ischaemic stroke versus transient ischaemic attack (1·37, 1·01–1·87). The presence of proximal internal carotid artery stenosis was also predictive of any 50–99% intracranial stenosis, independently of age (appendix p 11).

**Table 2:** Predictors of any symptomatic or asymptomatic 50–99% intracranial stenosis

		**Unadjusted risk predictors odds ratio (95% CI)**	**p value**	**Age-adjusted risk predictors odds ratio (95% CI)**	**p value**	**Multivariable risk predictors odds ratio (95% CI)**	**p value**
Age, per 10 years		1·71 (1·51–1·94)	<0·0001	..	..	1·60 (1·39–1·83)	<0·0001
Male sex		1·10 (0·83–1·45)	0·51	1·31 (0·98–1·76)	0·066	1·17 (0·87–1·58)	0·31
Hypertension		2·16 (1·60–2·91)	<0·0001	1·58 (1·15–2·15)	0·0040	1·34 (0·96–1·87)	0·081
Diabetes		1·53 (1·05–2·24)	0·027	1·48 (1·00–2·18)	0·050	1·19 (0·79–1·81)	0·41
Hyperlipidaemia		1·58 (1·19–2·11)	0·0020	1·39 (1·03–1·86)	0·029	1·04 (0·76–1·44)	0·79
Atrial fibrillation		1·87 (1·32–2·66)	<0·0001	1·34 (0·93–1·93)	0·12	1·18 (0·81–1·71)	0·39
Any previous vascular disease*		2·53 (1·89–3·38)	<0·0001	1·83 (1·35–2·49)	<0·0001	..	..
Previous stroke or transient ischaemic attack		2·14 (1·52–3·02)	<0·0001	1·72 (1·21–2·46)	0·0030	1·64 (1·13–2·38)	0·0090
Peripheral vascular disease		3·29 (1·93–5·63)	<0·0001	2·49 (1·43–4·33)	0·0010	1·94 (1·09–3·45)	0·024
Ischaemic heart disease		2·38 (1·66–3·40)	<0·0001	1·68 (1·16–2·44)	0·0060	1·34 (0·90–1·99)	0·18
Event type							
	Transient ischaemic attack	1·00 (ref)	..	1·00 (ref)	..	1·00 (ref)	..
	Minor ischaemic stroke	1·28 (0·96–1·71)	0·089	1·40 (1·04–1·88)	0·027	1·37 (1·01–1·87)	0·040

* Vascular disease includes ischaemic stroke, transient ischaemic attack, peripheral vascular disease, or ischaemic heart disease.

**Figure 2:**
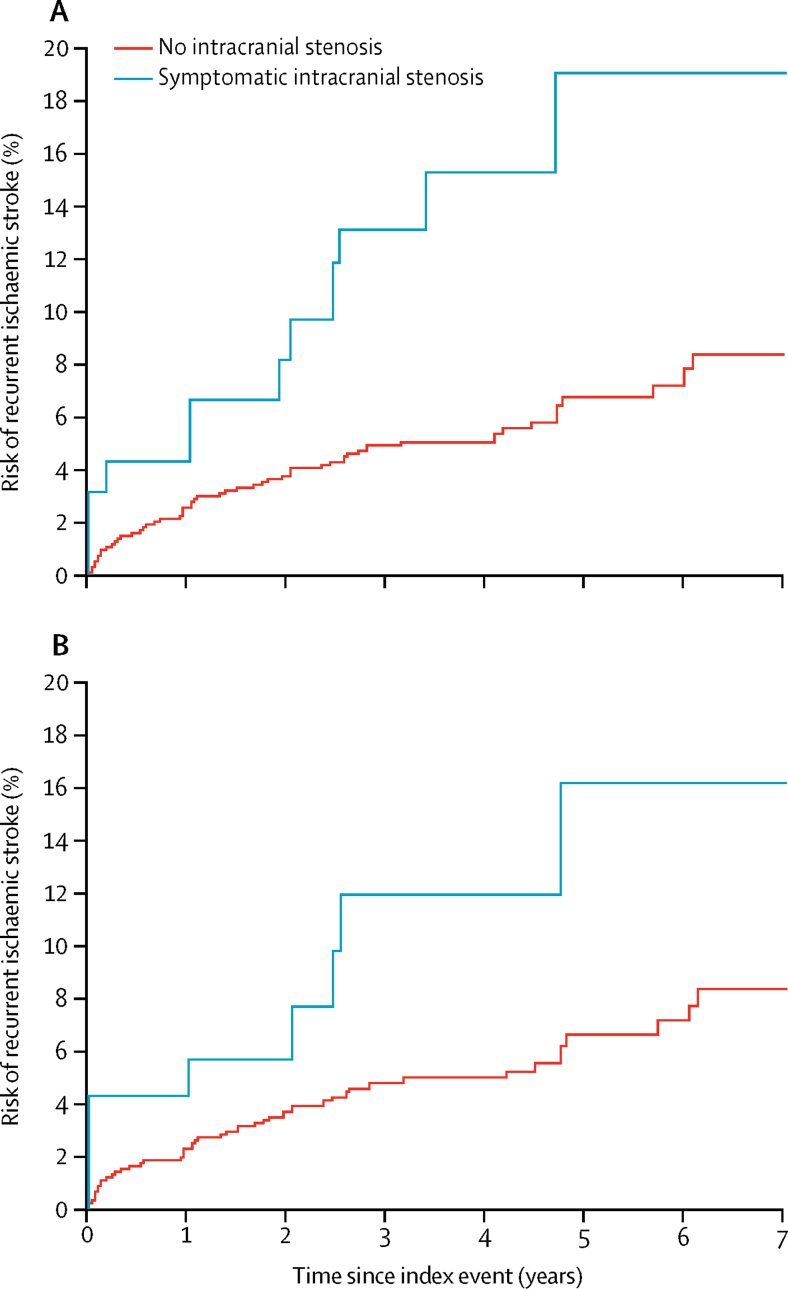
Risk of recurrent ischaemic stroke in patients with 50–99% symptomatic intracranial stenosis and those without intracranial stenosis Graphs show the 7-year risk of recurrent ischaemic stroke in all patients (A) and excluding those with atrial fibrillation or cardioembolic cause (B).

All patients had at least 6 months of follow-up. Stroke risk differed between patients with symptomatic 50–99% intracranial stenosis and no intracranial stenosis (figure 2). Of 94 patients with symptomatic 50–99% intracranial stenosis, 12 (12·8%) had recurrent ischaemic and two (2·1%) had intracerebral haemorrhages during median follow-up of 2·8 years (IQR 1·5–4·6). Of the 12 patients with recurrent ischaemic stroke, four patients had minor (NIHSS ≤3) and eight had major recurrent ischaemic strokes. On Cox regression there was no difference between patients with asymptomatic intracranial stenosis versus no intracranial stenosis in risk of recurrent ischaemic stroke, but risk was increased in patients with symptomatic intracranial stenosis (appendix p 12). This increased risk remained after adjustment for age, event type, and previous ischaemic vascular events (hazard ratio 1·43, 95% CI 1·04–1·96) and risks of any ischaemic vascular event and all-cause death were also increased (appendix p 12).

Characteristics of the 36 patients in OXVASC fulfilling the SAMMPRIS and VISSIT trial inclusion criteria differed (all p<0·05) from those in the SAMMPRIS trial non-stenting group (appendix p 13) in relation to age, ethnicity, and vascular risk factors (hypertension, diabetes, hyperlipidaemia, and current smoking), with similar trends also evident in comparison with VISSIT.

The 2-year risk of any recurrent ischaemic stroke in patients with symptomatic 70–99% intracranial stenosis in our cohort was 10·4% (95% CI 1·8–19·0). The absolute risk of same-territory ischaemic stroke during all follow-up was greater in patients with 70–99% compared with those with 50–69% symptomatic intracranial stenosis (22·9% [95% CI 6·0–39·8] *vs* 4·8% [0·0–11·3]).

In relation to the outcome definitions and follow-up durations reported in the trials (table 3), the 2-year risk of any stroke or death in OXVASC (22·7%, 95% CI 8·8–36·6) was similar to that in SAMMPRIS (19·8%, 15·1–25·6) and the 1-year risk of same-territory ischaemic stroke or transient ischaemic attack in OXVASC (13·9%, 2·5–25·3) was comparable with that in VISSIT (15·1%, 6·7–27·6). The long-term (beyond 1 year) rates of same-territory recurrent ischaemic stroke were similar in OXVASC (1·4 events per 100 patient-years) and SAMMPRIS (1·0 events per 100 patient-years). The risk of any recurrent ischaemic stroke in all patients (as reported in some previous studies; appendix pp 4–6) is greater than risk of any same-territory recurrent ischaemic stroke in patients with 50–99% and 70–99% symptomatic intracranial stenosis (appendix p 18).

**Table 3:** Comparison of the outcomes reported in the non-stenting medical treatment-only groups of the SAMMPRIS and VISSIT trials with comparable outcomes in the OXVASC cohort

	Any stroke*or death <30 days and same territory ischaemic stroke >30 days†		Any stroke*or death†		Any stroke*†		**Same territory ischaemic stroke or hard transient ischaemic attack (>2 days)**‡		**Same territory ischaemic stroke**‡		**Any territory hard transient ischaemic attack (>2 days)**‡	
	Events	Risk (95% CI)	Events	Cumulative risk (95% CI)	Events	Cumulative risk (95% CI)	Events	Cumulative risk (95% CI)	Events	Cumulative risk (95% CI)	Events	Cumulative risk (95% CI)
SAMMPRIS, n=227	34	14·1 % (10·1–19·4)	51	19·8% (15·1–25·6)	13	17·2% (12·9–22·9)	..	..	..	..	..	..
VISSIT, n=53	..	..	..	..	..	..	8	15·1% (6·7–27·6)	5	9·4% (3·1–20·7)	3	5·7% (1·2–15·7)
All OXVASC 50–99% symptomatic intracranial stenosis, n=94	8	9·0% (2·9–15·1)	21	23·4% (14·6–32·2)	9	10·8% (3·9–17·7)	11	12·0% (5·3–18·7)	5	5·5% (0·8–10·2)	7	7·6% (2·1–13·1)
OXVASC 50–99% symptomatic intracranial stenosis excluding atrial fibrillation, n=74	6	8·2% (1·9–14·5)	16	22·6% (12·8–32·4)	6	8·9% (2·0–15·8)	8	11·1% (3·9–18·4)	4	5·6% (0·3–11·1)	5	6·9% (1·0–12·8)
OXVASC 70–99% symptomatic intracranial stenosis fulfilling trial criteria, n=36	2	5·6% (0·0–13·0)	8	22·7% (8·8–36·6)	3	9·2% (0·0–19·2)	5	13·9% (2·5–25·3)	2	5·6% (0·0–13·0)	3	8·3% (0·0–17·3)

* Any stroke includes ischaemic stroke, intracerebral haemorrhage, or subarachnoid haemorrhage.

† SAMMPRIS 2-year outcome.

‡ VISSIT 1-year outcome.

Among the 74 patients without atrial fibrillation and 50–99% symptomatic intracranial stenosis, there was a high rate of compliance with antiplatelet and statin therapy up to 5 years of follow-up (appendix p 14). At baseline, 45 (60·8%) of 74 patients were on two or more antihypertensives, increasing to 18 (69·2%) of 26 patients at 5 years of follow-up (appendix p 14). Among 94 patients with 50–99% symptomatic intracranial stenosis, patients with recurrent stroke or who died during follow-up were older, had a greater burden of diabetes and intracranial stenosis, and higher 1-month systolic blood pressures than those without (appendix p 15).

## Discussion

To our knowledge, this is the first population-based study of predominantly Caucasian patients with minor ischaemic stroke or transient ischaemic attack and a high rate of intracranial imaging. We have found symptomatic or asymptomatic 50–99% intracranial stenosis in 241 (17·6%) patients in our population, with highest rates at older ages. Previous hospital-based studies done in predominantly Caucasian patients with transient ischaemic attack or stroke have reported a wide range of prevalence of symptomatic intracranial stenosis, with rates varying from 0·04% to 36·36%, probably reflecting in part differences in the definition of intracranial stenosis, imaging technique, inclusion criteria, and completeness of ascertainment (appendix pp 1–3).

The presence of symptomatic 50–99% intracranial stenosis was independently associated with an increased risk of recurrent ischaemic stroke which was higher for the 70–99% intracranial stenosis subgroup. The 2-year risk of any recurrent ischaemic stroke in patients with symptomatic 70–99% intracranial stenosis in OXVASC was 10·4% (95% CI 1·8–19·0). This risk was lower than that reported in earlier studies,^16^, ^27^ and prognosis was comparable to that in the medical treatment groups of the SAMMPRIS and VISSIT trials. Furthermore, the long-term (beyond 1 year) rates of same-territory recurrent ischaemic stroke were similar in OXVASC and SAMMPRIS. The risk of recurrent stroke in OXVASC was also dependent on the definition of the outcome (appendix p 18), which might explain some of the heterogeneity in risks reported in previous studies (appendix pp 4–6).

The impact of randomised controlled trials and systematic reviews depends on the external validity (or generalisability)—ie, the extent to which the results apply to a definable group of patients in a particular setting.^28^ Both SAMMPRIS and VISSIT had young cohorts, due to the exclusion of elderly patients, and reported lower than predicted recurrent event rates on medical treatment alone. Our findings in a population-based cohort, including many older patients, nevertheless supports the external validity of the trials.

The low risk of stroke on medical treatment alone in SAMMPRIS has been suggested to be due to the intensity of risk factor management.^17^, ^29^ The patients in OXVASC also received intensive medical management, similar to that of both SAMMPRIS and VISSIT. This consisted of dual anti-platelet therapy (aspirin and clopidogrel) for the first month with clopidogrel or aspirin monotherapy thereafter, high-intensity statin treatment and ambulatory monitoring of blood pressure (target of <130/80 mm Hg). Patients were also given advice on smoking cessation, exercise, and diet and were regularly followed-up by study research nurses to ensure medication compliance and adequate blood pressure control. For example, in patients with 50–99% symptomatic intracranial stenosis, systolic blood pressure was reduced by about 20 mm Hg between baseline and 1-month follow-up.

Routine screening for extracranial internal carotid artery stenosis as secondary prevention of stroke is supported by international guidelines,^10^, ^30^ but there is no consensus on the utility of routine screening for intracranial stenosis. Although the increased use of intracranial vascular imaging in the treatment of major acute stroke sometimes identifies patients with intracranial stenosis, patients with transient ischaemic attack or minor stroke are often not screened. Our results show that although the stroke risk is similar to that in SAMMPRIS and VISSIT, patients with symptomatic intracranial stenosis are nevertheless a high-risk subgroup even when treated according to guidelines. Although the SAMMPRIS and VISSIT trials provided no evidence to support a role for percutaneous stenting for symptomatic intracranial stenosis, the high stroke risk might justify routine screening to tailor risk factor management. For example, as intensive lipid-lowering with monoclonal antibodies becomes available, the high costs are likely to limit treatment to subgroups of patients with a high risk of atherosclerotic disease. There is also some evidence that combinations of antithrombotic agents might also be effective in reducing stroke risk in patients with an increased risk of atherosclerotic disease.^31^ Moreover, recruitment into future trials in patients with intracranial stenosis will be difficult if potentially eligible patients are not identified. Additionally, in patients with apparently cryptogenic transient ischaemic attack or stroke, knowledge of a symptomatic intracranial stenosis is likely to motivate both patient and physician to comply with intensive medical treatment, particularly given the resistance on the part of some patients to take statins^32^, ^33^ and that some clinicians are reluctant to prescribe lipid-lowering drugs in the very elderly, in whom we found high rates of intracranial stenosis.

The strengths of our study include its large, population-based nature, with a large number of events captured in the target population, a long period of follow-up, and intensive medical management; nearly 90% of eligible patients underwent intracranial vascular imaging of some kind, the majority receiving MR angiography. We chose the most commonly used definition of intracranial stenosis (50–99% luminal stenosis, further subcategorised to 70–99% for trial comparison) and showed good inter-rater reliability. However, our study also had some limitations. First, our findings do not apply to patients with major stroke. However, 90% of all recurrent strokes occur after a transient ischaemic attack or minor stroke^34^ and the previous trials (SAMMPRIS and VISSIT)^1^, ^2^ of stenting for intracranial stenosis also predominantly recruited patients with transient ischaemic attack or minor stroke. Second, although our patients were followed-up regularly by study nurses and clinicians offering similar lifestyle advice and risk factor management to that in SAMMPRIS and VISSIT trials,^1^, ^2^ rates of medication compliance and risk factor control are likely to have been higher than in patients in normal clinical practice. Third, although two-thirds of patients in our study received our first preference of MR angiography, other imaging methods had to be used when MR angiography was contraindicated, principally CT angiography, which has different sensitivity and specificity for detecting intracranial stenosis. Although we did find differences in the detection rates between MR and CT angiography (appendix p 10), CT angiography was used in a subgroup of older patients with contraindications to MRI (eg, pacemakers) and with a greater burden of vascular risk factors. Fourth, time-of-flight MR angiography is prone to artefact because of flow abnormalities—low flow might mimic stenosis and high flow through stenosis might underestimate its degree.^35^ However, we used a combination of contrast enhanced and time-of-flight MR angiography to improve the specificity of intracranial stenosis detection, and MR angiography is commonly used clinically to detect intracranial stenosis owing to the unacceptable risks of catheter angiography for screening. The SAMMPRIS and VISSIT trials^1^, ^2^ only included patients with high-grade 70–99% symptomatic intracranial stenosis as determined by catheter angiography. Although catheter angiography is more accurate in grading intracranial stenosis than non-invasive angiography, there are associated risks and it cannot now be used for routine screening or for research. Non-invasive angiography was therefore the only suitable method of answering the study questions, and the increased risk of same-territory recurrent stroke seen in those with higher grade intracranial stenosis supports the accuracy of non-invasive angiography. Finally, although the OXVASC cohort was older, there were fewer vascular comorbidities than in the SAMMPRIS population. This difference was likely due to differing patient selection: SAMMPRIS required at least one vascular risk factor in patients younger than 50 years. Moreover, both SAMMPRIS and VISSIT had upper age limits for eligibility, and younger-onset vascular disease tends to be associated with more vascular risk factors.

In conclusion, intracranial stenosis is prevalent in older Caucasian patients with minor ischaemic stroke or transient ischaemic attack, and the risk of recurrent stroke following symptomatic intracranial stenosis was consistent with the two randomised controlled trials (SAMMPRIS and VISSIT) in younger cohorts. Given the likely generalisability of the trials' results to the broader patient population, routine screening for intracranial stenosis would not be justified to identify candidates for stenting, but intracranial stenosis does identify those with a higher risk of atherosclerotic disease who may require tailored risk factor management and recruitment to future clinical trials.

## Data sharing

Requests for access to the data reported in this paper will be considered by the corresponding author.
