# Supplementary material for: Prevalence, predictors, and prognosis of symptomatic intracranial stenosis in patients with transient ischaemic attack or minor stroke: a population-based cohort study
Source: Lancet Neurol. 2020 May;19(5):413–21. doi: 10.1016/S1474-4422(20)30079-X (PMC7116132; doi:10.1016/S1474-4422(20)30079-X)
Supplement: Supplementary appendix [file mmc1.pdf]

THE LANCET
Neurology

Supplementary appendix

This appendix formed part of the original submission and has been peer reviewed. We post it as supplied by the authors.

Supplement to: Hurford R, Wolters FJ, Li L, et al. Prevalence, predictors, and prognosis of symptomatic intracranial stenosis in patients with transient ischaemic attack or minor stroke: a population-based cohort study. *Lancet Neurol* 2020; **19:** 413–21.

Supplementary Material

Supplementary Table 1a: Prevalence of ICS in medically treated Caucasian TIA/ stroke patients

Study	Location	Sample size	Mean age (years)	Caucasian (%)	IS/ TIA	Definition of ICS		Prevalence of ICS	
						Screening imaging modality	Criteria	Any ICS n (%)	Symptomatic ICS n (%)
All ICS									
Sacco (NOMASS) 1995[1]	USA	82	70	100	IS	TCD	Velocity criteria	-	1.0%*
Wityk 1996[2]	USA	108	75	100	IS/TIA	TCD/MRA/CA	Velocity criteria or 50-100% stenosis	26 (24.0)	~9.0%*
Thijs 2000[3]	USA	1344	-	-	IS/TIA	CA/TCD/MRA	Velocity criteria or 50-99% stenosis	54 (4.0)	36 (2.7)
Weimar 2006[4]	Germany	4,157	67	-	IS/TIA	TCD/MRA/CTA/CA	Velocity criteria or 50-100% stenosis	1259 (30.3)	611 (14.7)
Meseguer 2010[5]	France	1,823	61	-	TIA	TCD	Velocity criteria	161 (8.8)	67 (3.7)
Weber 2010[6]	Germany	13,584	67	-	IS/TIA	TCD/MRA	Velocity criteria or 50-99% stenosis	736 (5.4)	304 (2.2)
Lau 2013[7]	USA	539	66	83	IS/TIA	CTA	Any grade of stenosis	212 (39.3)	176 (32.7)
Holzer 2009[8]	Germany	163	63	100	TIA	TCD	Velocity criteria	15 (9.2)	-

Homburg 2011[9]	Netherlands	786	62	90	IS/TIA	CTA	50-100% stenosis	77 (9.8)	18 (2.3)
Von Weitzel-Mudersbach 2012[10]	Denmark	195	66	100	TIA	TCD	Velocity criteria	24 (12.3)	16 (8.2)
Amarenco 2012[11]	France	1,679	64	-	TIA	TCD	Velocity criteria	-	85 (5.0)
Ovesen 2013[12]	Denmark	652	67	95	IS/TIA	CTA	≥30% stenosis	101 (15.5)	3 (0.04)
Ssi-Yan-Kai 2013[13]	France	129	64	-	IS/ TIA	MRA	50-100% stenosis	-	16 (12.4)
Wolff 2014[14]	France	159	37	99	Young IS (age 18-45)	MRA/CTA/DSA	≥50% stenosis	-	49 (31.2)
Logallo 2014[15]	Norway	607	73	-	IS/TIA	TCD/MRA/CTA	Velocity criteria or any degree stenosis	69 (11.4)	45 (7.4)
Tsivgoulis 2014[16]	Greece	467	58	98	IS/TIA	TCD	Velocity criteria	51 (10.9)	46 (9.8)
Gouveia 2014[17]	Portugal	1,302	72	-	IS/TIA	TCD/CTA	Velocity criteria or ≥50% stenosis	158 (12.1)	75 (5.8)
Baracchini 2016[18]	Italy	1,134	71	97	IS	TCD	Velocity criteria	-	99 (8.7)
Hoshino 2018[19]	France	403	62	-	IS	TCD/MRA/CTA	Velocity criteria or 50-100% stenosis	146 (36.2)	72 (17.9)
Uchiyama 2019[20]	France	3317	66	78	IS/TIA	TCD/MRA/CTA	Velocity criteria or 50-100% stenosis	424 (12.8)	-
Anterior circulation ICS only									

Kappelle (NASCET)1999[21]	USA	2589	66	95	IS/ TIA	CA	50-100% ACA, MCA, intracranial ICA stenosis	-	14[§] (0.5)
von Sarnowski [¥] 2013[22]	Pan-European	1,561	46	-	Young IS/ TIA (age 18-55)	TCD	Velocity criteria	184 (11.8)	137 (8.8)
Mattioni 2014[23]	Netherlands	220	65	-	IS/TIA	CTA	Intracranial ICA and MCA any degree stenosis or occlusion	85 (38.6)	80[§] (36.4)
Posterior circulation ICS only									
Marquardt 2009[24]	UK	141	69	95	Vertebrobasilar IS/TIA	MRA	≥50% intracranial VA or BA stenosis	-	14 (9.9)
Klein 2010[25]	France	41	66	-	Pontine IS	MRA	≥30% BA stenosis	-	7 (17.1)
von Sarnowski [¥] 2013[22]	Pan-European	1,511	46	-	Young IS/ TIA (age 18-55)	TCD	Velocity criteria	75 (5.0)	45 (3.0)

[*]N not reported, [§]Ipsilateral to symptomatic ICA stenosis, [¥] study reports anterior and posterior circulation ICS separately without noting duplicate patients hence are reported separately in this table. Prevalence of stenoses reported with different denominators for anterior and posterior circulation. IS= ischaemic stroke, ICS= intracranial stenosis, CA= catheter angiography, TCD= transcranial Doppler, MRA= magnetic resonance angiography, CTA= computed tomography angiography, DSA= digital subtraction angiography.

Supplementary Table 1b: Risk of recurrent ischaemic stroke in Caucasian TIA/ stroke patients due to symptomatic ICS receiving medical treatment only.

Study	Location	Sample size	Mean age (years)	Caucasian (%)	IS/ TIA	Definition of ICS		Prognosis of ICS		
						Screening imaging modality	Criteria	Mean follow-up (months)	Any IS n (%)	Same territory IS n (%)
All ICS										
Thijs 2000[3]	USA	52	67	65	IS/TIA	CA/TCD/MRA	Velocity criteria or 50-99% stenosis	17	6 (11.5)	-
Chimowitz (WASID) 2005[26]	USA	280*	63	58	IS/TIA	TCD/MRA/ CTA	≥50% stenosis	22	57 (20.4)	42 (15.0)
Mazighi 2006[27]	France	102	63	97	IS/TIA	MRA/CTA/ DSA	50-99% stenosis	23	-	14 (13.7)
Weimar 2006[4]	Germany	272	67	-	IS/TIA	TCD/MRA/ CTA/CA	Velocity criteria or 50-100% stenosis	-	26 (9.6) at 1y	-
Samaniego 2009[28]	USA	58	65	83	IS/TIA	MRA/CTA/ DSA	Undefined	14	3 (5.1)	-
Weber 2010[6]	Germany	197	65	-	IS/TIA	TCD/MRA	Velocity criteria or 50-99% stenosis	24	23.3% at 3y	-
Kozak 2011[29]	USA	25	61	72	IS/TIA	CA	50-99% stenosis	16	-	11 (44.0)
Nahab 2013[30]	USA	22	66	59	IS/TIA	CA/CTA	50-99% stenosis	14	0	0
Gouveia 2014[17]	Portugal	72	73	-	IS/TIA	TCD/CTA	Velocity criteria or ≥50% stenosis	14	-	14 (19.4)

Derdeyn (SAMMPRIS) 2014[31]	USA	227	60	71	IS/TIA	CA	70-99% stenosis	32	-	31 (13.7)
Zaidat (VISSIT) 2015[32]	USA	53	62	72	IS/TIA	CA	70-99% stenosis	-	-	5 (9.4) at 1y
Caliandro 2018[33]	Italy	48	65	-	IS/TIA	TCD	Velocity criteria	-	-	5[¥] (10.4) at 1y
Hoshino 2018[19]	France	72	65	-	IS	TCD/MRA/ CTA	Velocity criteria or 50-100% stenosis	-	9[¥] (13.2) at 4y	-
Uchiyama (TIAregistry) 2019[20]	France	4,238	66	78	IS/TIA	TCD/MRA/ CTA	Velocity criteria or 50-100% stenosis	-	194 (4.8) at 1y	-
Anterior circulation ICS only										
Hinton 1979[34]	USA	16	62	-	IS/TIA	CA	40-100% MCA stenosis	36	-	2 (12.5)
EC/IC bypass study group 1985[35]	Worldwide	714	56	-	IS/TIA	CA	Any degree of MCA and intracranial ICA stenosis	56	-	~10.0% at 1y
Wechler 1986[36]	USA	12	62	-	IS/TIA	CA	50-100% carotid siphon stenosis	51	-	3 (25.0)
Kappelle (NASCET) 1999[21]	USA	2589	66	93	IS/TIA	CA	50-100% ACA, MCA, intracranial ICA stenosis	-	-	19.4-45.7%[§] at 3y
Arenillas 2001[37]	Spain	40	62	-	IS/TIA	TCD	MCA velocity criteria	27	-	8 (20.0)
Kern 2005[38]	Germany	46	57	-	IS/TIA	TCD	Velocity criteria	-	-	11[¥] (23.9)
Posterior circulation ICS only										

Moufarrijj 1986[39]	USA	44	~58	-	Vertebrobasilar IS/TIA	CA	≥50% intracranial VA or BA stenosis	73	-	5 (11.4)
Pessin 1987[40]	USA	9	62	56	Vertebrobasilar IS/TIA	CA	≥40% distal BA stenosis	21	-	1 (11.1)
Pessin 1987[41]	USA	6	70	-	PCA territory IS/TIA	CA	≥50% PCA stenosis	9	0	0
Qureshi 2003[42]	USA	102	64	54	Vertebrobasilar IS/TIA	CA/MRA	50-99% intracranial VA or BA stenosis	15	14 (13.7)	8 (7.8)
Markus (VIST) 2017[43]	UK	88	67	-	Vertebrobasilar IS/TIA	MRA/CTA/ DSA	≥50% intracranial VA stenosis	29	-	4 (4.6)

**patients randomised to receive aspirin,* [§] *medical arm of NASCET*, [¥] *ischaemic stroke and TIA*. IS= ischaemic stroke, TIA= transient ischaemic attack, ICS= intracranial stenosis, CA= catheter angiography, TCD= transcranial Doppler, MRA= magnetic resonance angiography, CTA= computed tomography angiography, DSA= digital subtraction angiography, MCA middle cerebral artery, ICA= internal carotid artery, VA= vertebral artery, BA= basilar artery, PCA= posterior cerebral artery

Supplementary Table 2: Standard anatomical landmarks used to locate extra- and intracranial stenoses.

Vessel	Anatomical landmark
Subclavian artery	Right – from the brachiocephalic trunk (left - from the aortic arch) to the lateral border of the first rib.
Common carotid artery	Right – from the brachiocephalic trunk (left - from the aortic arch) to the terminal internal and external carotid artery branches.
Cervical internal carotid artery (ICA)	Left and right. Cervical segment – terminal branch of common carotid artery to petrous portion of temporal bone.
Distal internal carotid artery (ICA)	Left and right. C2 to C7 – petrous portion of temporal bone to the terminal branching of ACA and MCA.
Middle cerebral artery (MCA) M1	Left and right terminal bifurcation of ICA until bifurcation to superior or inferior divisions.
Middle cerebral artery (MCA) M2	From the main bifurcation of MCA coursing posterosuperiorly within the insula cleft to circular sulcus of insula.
Anterior cerebral artery (ACA)	Left and right branches of ICA. A1, A2 and anterior communicating subdivisions considered as one.
Posterior communicating artery (Pcomm)	Left and right from the distal ICA (prior to the ACA and MCA bifurcation) to the PCA P1 segment.
Posterior cerebral artery (PCA) P1	Left and right terminal branches of the basilar artery to the Pcomm in interpeduncular cistern.
Posterior cerebral artery (PCA) P2	Left and right. From P1 Pcomm junction to quadrigeminal cistern.
Basilar artery (BA)	Confluence of left and right V4 to terminal bifurcation of left and right PCA.
Vertebral artery (VA) V1	Left and right – origin from subclavian arteries to transverse foramen of C6 vertebrae.
Vertebral artery (VA) V2	Left and right – from transverse foramen of C6 vertebrae to transverse foramen of C2.
Vertebral artery (VA) V3	Left and right – from transverse foramen of C2 to dura.
Vertebral artery (VA) V4	Left and right – from dura to basilar artery formation (or termination as posterior inferior cerebellar artery).

Supplementary Table 3: Details of imaging received by study patients and reasons for non-imaging.

**Imaging modality**	**Patients (%)**<br>**N= 1579**
**Intracranial vascular imaging**	
Magnetic resonance angiography	1034 (65.5)
Computed tomography angiography	253 (16.0)
Transcranial Doppler ultrasound	81 (5.2)
**Extracranial vascular imaging only**	
Carotid Doppler ultrasound	154 (9.8)
**No vascular imaging**	57 (3.6)
Known atrial fibrillation	*25*
Previously investigated or imaged elsewhere	*8*
Other clinical or logistical issue	*24*

Supplementary Table 4: Baseline characteristics of study population, stratified by intracranial vascular imaging.

	Intracranial vascular imaging (N= 1368)	***No intracranial vascular imaging (N= 211)***	**p-value**
Mean age (SD)	69.2 (13.9)	76.5 (12.1)	<0.0001
Male sex (%)	700 (51.2)	110 (52.1)	0.82
Caucasian (%)	1291 (94.4)	201 (95.3)	0.75
Hypertension (%)	754 (55.1)	147 (69.7)	<0.0001
Diabetes mellitus (%)	183 (13.4)	48 (22.7)	0.0004
Hyperlipidaemia (%)	466 (34.1)	79 (37.4)	0.35
Current smoker (%)	193 (14.1)	25 (11.8)	0.45
Atrial fibrillation (%)	204 (14.9)	57 (27.0)	<0.0001
Any vascular disease[§] (%)	373 (27.3)	106 (50.2)	<0.0001
History of stroke or TIA (%)	204 (14.9)	76 (36.0)	<0.0001
PVD (%)	61 (4.5)	17 (8.1)	0.038
IHD (%)	179 (13.1)	52 (24.6)	0.0005
Event type			
TIA (%)	892 (65.2)	108 (51.2)	<0.0001
Minor stroke (%)	476 (34.8)	103 (48.8)	
TOAST classification			
Cardioembolic (%)	210 (15.4)	69 (32.7)	
Atherosclerotic (%)	181 (13.2)	21 (10.0)	
Undetermined (%)	543 (39.7)	35 (16.6)	0.15
Lacunar (%)	133 (9.7)	24 (11.4)	
Multiple/unknown/other (%)	301 (22.0)	62 (29.3)	
Vascular territory			
Carotid (%)	716 (52.3)	148 (70.1)	
Vertebrobasilar (%)	504 (36.8)	37 (17.5)	<0.0001
Uncertain/ both (%)	148 (10.8)	26 (12.3)	

PVD= peripheral vascular disease, IHD= ischaemic heart disease. [§]vascular disease = ischaemic stroke/ TIA, PVD or IHD.

Supplementary Table 5: Distribution of any 50-99% intracranial stenosis (ICS) and extracranial vascular stenosis stratified by magnetic resonance angiography (MRA) and computed tomography angiography (CTA).

	**Affected vessels / total number of vessels imaged (%)¶**	**MRA, 1034 patients**	**CTA, 253 patients**	**p-value**
**Intracranial vessels**				
Distal ICA	84/ 2458 (3.4)	49/ 1954 (2.5)	35/ 504 (6.9)	<0.0001
ACA	29/ 2565 (1.1)	28/ 2059 (1.4)	1/ 506 (0.2)	0.031
MCA (M1+M2)	98/ 2573 (3.8)*	84/ 2063 (4.1)	10/ 506 (2.0)	0.048
PCA (P1+P2)	93/ 2559 (3.6)	83/ 2053 (4.0)	10/ 506 (2.0)	0.024
PComm	3/ 847 (0.4)	3/ 685 (0.4)	0/ 162 (0)	0.99
Basilar	13/ 1285 (1.0)	11/ 1032 (1.1)	2/ 253 (0.8)	0.99
Vertebral (V4)	74/ 2557 (2.9)	53/ 2051 (2.6)	21/ 506 (4.2)	0.072
**Extracranial vessels**				
Subclavian	76/ 2424 (3.1)	69/ 1944 (3.6)	7/ 480 (1.5)	0.018
Common carotid	34/ 2900 (1.2)¤	11/ 1960 (0.6)	22/ 491 (4.5)	<0.0001
Proximal ICA	273/ 2867 (9.5)¥	132/1960(6.7)	67/491 (13.6)	<0.0001
Vertebral (V1-3)	236/ 2447 (9.6)	200/1949 (10.3)	36/469 (7.7)	0.10

*ICA= internal carotid artery; ACA = anterior cerebral artery; MCA = medial cerebral artery; PCA = posterior cerebral artery; PComm = posterior communicating artery. ¶A single ICS affecting two contiguous vessel segments are counted as two. *Including 4 MCA ICS detected by transcranial Doppler. ¤ Including carotid Doppler ultrasound 1 / 449; ¥including carotid Doppler ultrasound 74 / 416.*

Supplementary Table 6: The association between the presence of proximal extracranial internal carotid artery (ICA)* stenosis and intracranial stenosis (ICS) stratified by age.

N (%)	ICS	No ICS
**All ages**		
ICA stenosis	*57 (32.0)*	*121 (68.0)*
No ICA stenosis	*167 (15.1)*	*936 (84.9)*
OR (95% CI)	2.64 (1.85-3.77), p<0.0001	
**<70 years**		
ICA stenosis	*8 (18.6)*	*35 (81.4)*
No ICA stenosis	*38 (7.3)*	*482 (92.7)*
OR (95% CI)	2.90 (1.26- 6.69), p= 0.013	
**≥70 years**		
ICA stenosis	*49 (36.3)*	*86 (62.7)*
No ICA stenosis	*129 (22.1)*	*454 (77.9)*
OR (95% CI)	2.01 (1.34- 3.00), p=0.0010	

**17 patients did not have extracranial ICA imaging. ICA= internal carotid artery, OR= odds ratio; CI= confidence interval*

Supplementary Table 7: Risk of outcomes in the OXVASC cohort categorised by the presence of 50-99% symptomatic or asymptomatic intracranial stenosis (ICS) versus no ICS.

	**ICS Event/ total**	**No ICS Event/ total**	**Unadjusted HR (95% CI)**	**p-value**	**Adjusted HR§ (95% CI)**	**p-value**
**Asymptomatic ICS**						
Ischaemic stroke	8/ 147	57/ 1108	1.09 (0.52- 2.28)	0.82	0.76 (0.36- 1.62)	0.48
Death	28/ 147	115/ 1108	1.96 (1.30- 2.96)	0.001	1.05 (0.69- 1.59)	0.83
Ischaemic vascular event*	11/ 147	76/ 1108	1.10 (0.58- 2.06)	0.87	0.73 (0.38- 1.40)	0.34
**Symptomatic ICS**						
Ischaemic stroke	12/ 94	57/ 1108	1.63 (1.19- 2.22)	0.002	**1.43 (1.04- 1.96)**	0.027
Death	24/ 94	115/ 1108	1.65 (1.33- 2.06)	<0.0001	**1.29 (1.03-1.62)**	0.024
Ischaemic vascular event*	15/ 94	76/ 1108	1.56 (1.18- 2.05)	0.002	**1.35 (1.02- 1.79)**	0.038

*Patients with intracranial vessel occlusion (n=19) were excluded. *Isch vascular event= any of ischaemic stroke, peripheral vascular disease or ischaemic heart disease.*
§adjusted for age, event type and prior ischaemic vascular event.

Supplemental Table 8: Baseline characteristics of patients with 50-99% and 70-99% symptomatic ICS in the OXVASC cohort, compared with the no-stenting medical management only arms of SAMMPRIS (Derdeyn *et al.*[31]) and VISSIT (Zaidat *et al.*[32]).

**Characteristic**	**OXVASC** medical management 50-99% ICS[¶] **(N= 66)**	**OXVASC** medical management 70-99% ICS[¶] **(N= 36)**	**SAMMPRIS** medical management only arm **(N= 227)**	**p-value**[*]	**VISSIT** medical management only arm **(N= 53)**	**p-value**[§]
Mean age (SD)	73.0 (14.1)	70.5 (15.8)	59.5 (11.8)	<0.0001	61.8 (12.8)	0.0046
Male sex (%)	40 (60.6)	26 (72.2)	145 (63.9)	0.12	32 (60.4)	0.12
Caucasian (%)	62 (93.9)	36 (100.0)	162 (71.4)	<0.0001	38 (71.7)	<0.0001
Hypertension (%)	44 (66.7)	22 (61.1)	203 (89.4)	0.0002	43 (81.1)	0.10
Diabetes mellitus (%)	11 (16.7)	6 (16.7)	103 (45.4)	0.0037	20 (37.7)	0.12
Hyperlipidaemia (%)	26 (39.4)	13 (36.1)	202 (89.0)	<0.0001	32 (60.4)	0.064
PVD (%)	8 (11.6)	4 (11.1)	-	-	5 (9.4)	0.75
IHD (%)	13 (19.7)	6 (16.7)	59 (26.0)	0.34	12 (22.6)	0.80
Current smoker (%)	11 (16.7)	6 (16.7)	69 (30.4)	0.027	12 (22.6)	0.31
Prior stroke/ TIA (%)	19 (28.8)	9 (25.0)	58 (25.6)	0.53	-	-
Event type						
TIA (%)	36 (54.5)	22 (61.1)	75 (33.0)	0.003	22 (41.5)	0.071
Stroke (%)	30 (45.5)	14 (38.9)	152 (67.0)		34 (64.2)	
Vascular territory						
Carotid (%)	36 (54.5)	22 (61.1)	154 (67.9)	0.16	-	-
Vertebrobasilar (%)	30 (45.5)	14 (38.9)	73 (32.2)			

PVD= peripheral vascular disease, IHD= ischaemic heart disease.

[¶]*Otherwise patients fulfil trial inclusion criteria. Of 94 patients with 50-99% symptomatic ICS, 58 patients did not fulfil trial inclusion criteria (30 50-69% ICS, 20 coexistent atrial fibrillation, 4 bilateral vertebral artery ICS, 1 received percutaneous stenting, 1 medullary infarct with vertebral artery ICS and 2 tandem stenoses).*[*]*OXVASC 70-99% vs SAMMPRIS,* [§]*OXVASC 70-99% vs VISSIT*

Supplementary Table 9: Number of 50-99% symptomatic ICS patients (excluding those with atrial fibrillation) compliant with therapy during follow-up.

	**Baseline**	**1 month**	**6 months**	**1 year**	**5 years**
No antihypertensives (%)	12 (16.2)	9 (12.9)	10 (15.3)	9 (14.1)	6 (23.1)
1 antihypertensive (%)	17 (23.0)	12 (17.1)	12 (18.5)	2 (23.4)	2 (7.7)
≥2 antihypertensives (%)	45 (60.8)	49 (70.0)	43 (66.2)	40 (62.5)	18 (69.2)
Antiplatelet therapy (%)	74 (100)	69 (98.6)	61 (93.8)	57 (89.1)	25 (96.2)
Statin therapy (%)	65 (87.8)	65 (92.9)	59 (90.8)	58 (90.6)	23 (88.5)
Total number of patients	74	70	65	64	26

Supplementary Table 10: characteristics of patients with 50-99% symptomatic ICS

	**All symptomatic ICS patients N= 94**	Patients with recurrent stroke or death N= 35	Patients without recurrent stroke or death N= 59	**p-value**
Mean age (SD)	73.7 (13.5)	78.7 (12.7)	70.7 (13.2)	0.0049
Male (%)	59 (62.8)	21 (60.0)	38 (64.4)	0.83
Hypertension (%)	64 (68.1)	29 (82.9)	53 (59.3)	0.35
Hypertensive* at baseline (%)	79 (84.0)	26 (74.3)	53 (89.8)	0.078
Diabetes (%)	20 (21.3)	12 (34.3)	8 (13.6)	<0.0001
Hyperlipidaemia (%)	40 (42.6)	17 (48.6)	23 (39.0)	0.40
IHD (%)	22 (23.4)	9 (25.7)	13 (22.0)	0.80
PVD (%)	13 (13.8)	5 (14.3)	8 (13.6)	0.99
Current smoker (%)	11 (11.7)	5 (14.3)	6 (10.2)	0.74
Atrial fibrillation (%)	20 (21.3)	8 (22.9)	11 (18.6)	0.79
Prior stroke/ TIA (%)	22 (23.4)	9 (25.7)	13 (22.0)	0.80
Mean baseline SBP (SD)	154.9 (23.3)	149.1 (25.2)	158.0 (21.8)	0.074
Mean 1 month SBP (SD)	136.9 (18.5)	143.0 (20.0)	133.6 (17.0)	0.017
Intracranial stenoses				
Mean per patient (SD)	2.20 (1.6)	2.66 (2.0)	1.93 (1.3)	0.035
Ant circulation¥ (%)	63 (67.0)	28 (80.0)	35 (59.3)	0.042
Post circulation¥ (%)	63 (67.0)	23 (65.7)	40 (67.8)	0.99

**Systolic blood pressure >130/80mmHg. ¥Any anterior or posterior intracranial stenosis. SD= standard deviation; IHD= ischaemic heart disease; PVD= peripheral vascular disease; SBP= systolic blood pressure (mmHg); DBP= diastolic blood pressure (mmHg); ant= anterior; post= posterior.*

Supplementary Figure 1a: Flow diagram of systematic review inclusion/ exclusion for prevalence of ICS in Caucasian TIA/ stroke patients and Medline and Embase search terms

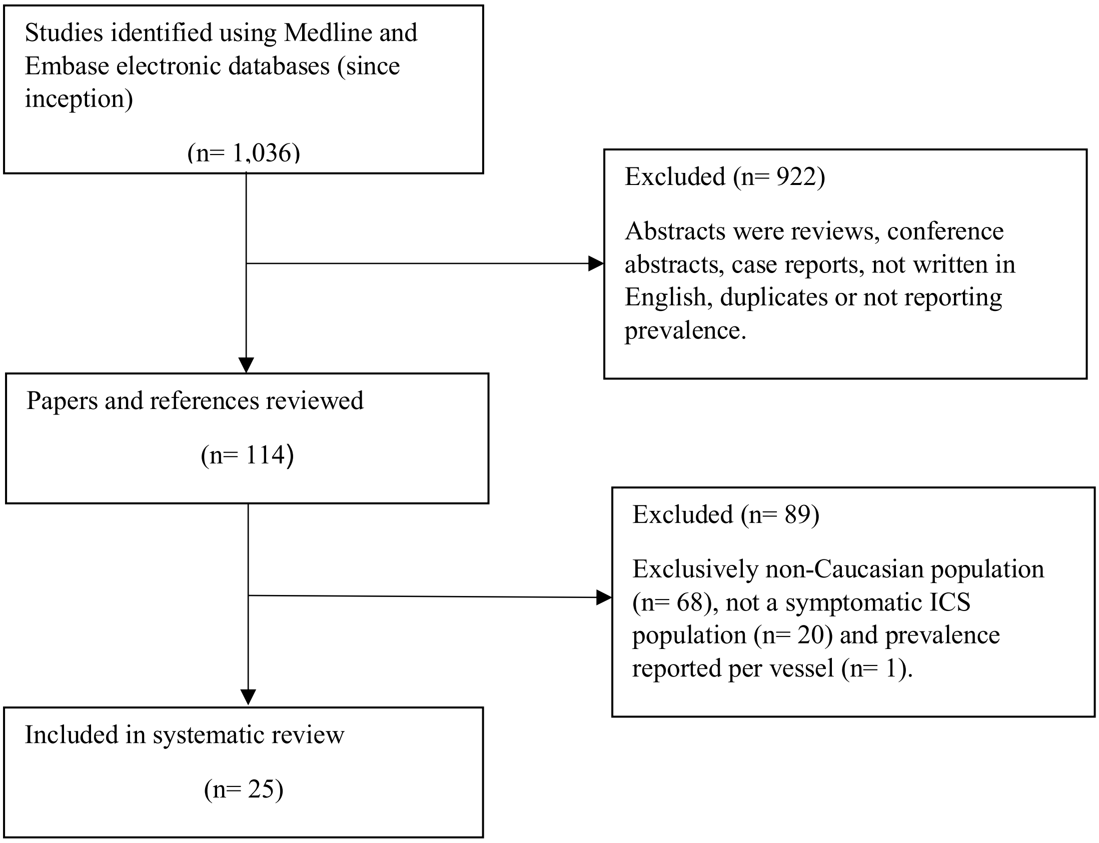

Medline search terms

1. Intracranial arteriosclerosis/
2. (intracranial adj3 stenos*).ti,ab.
3. (intra-cranial adj3 stenos*).ti,ab.
4. (intracranial adj3 arteriosclero*).ti,ab.
5. (intracranial adj3 atherosclero*).ti,ab.
6. ((brain or cerebr*) adj3 atherosclero*).ti,ab.
7. ((brain or cerebr*) adj3 arteriosclero*).ti,ab.
8. 1 or 2 or 3 or 4 or 5 or 6 or 7
9. Prevalence/
10. Prevalen*.ti,ab.
11. 9 or 10
12. 8 and 11

Embase search terms

1. *prevalence/
2. Prevalen*.ti,ab.
3. 1 or 2
4. *brain atherosclerosis/
5. (intracranial adj3 stenos*).ti,ab.
6. (intra-cranial adj3 stenos*).ti,ab.
7. (intracranial adj3 arteriosclero*).ti,ab.
8. (intracranial adj3 atherosclero*).ti,ab.
9. ((brain or cerebr*) adj3 atherosclero*).ti,ab.
10. ((brain or cerebr*) adj3 arteriosclero*).ti,ab.
11. 4 or 5 or 6 or 7 or 8 or 9 or 10
12. 3 and 11
13. Conference*.pt.
14. 12 not 13

Supplementary Figure 1b: Flow diagram of systematic review inclusion/ exclusion for prognosis medically treated Caucasian TIA/ stroke patients with symptomatic ICS and Medline and Embase search terms

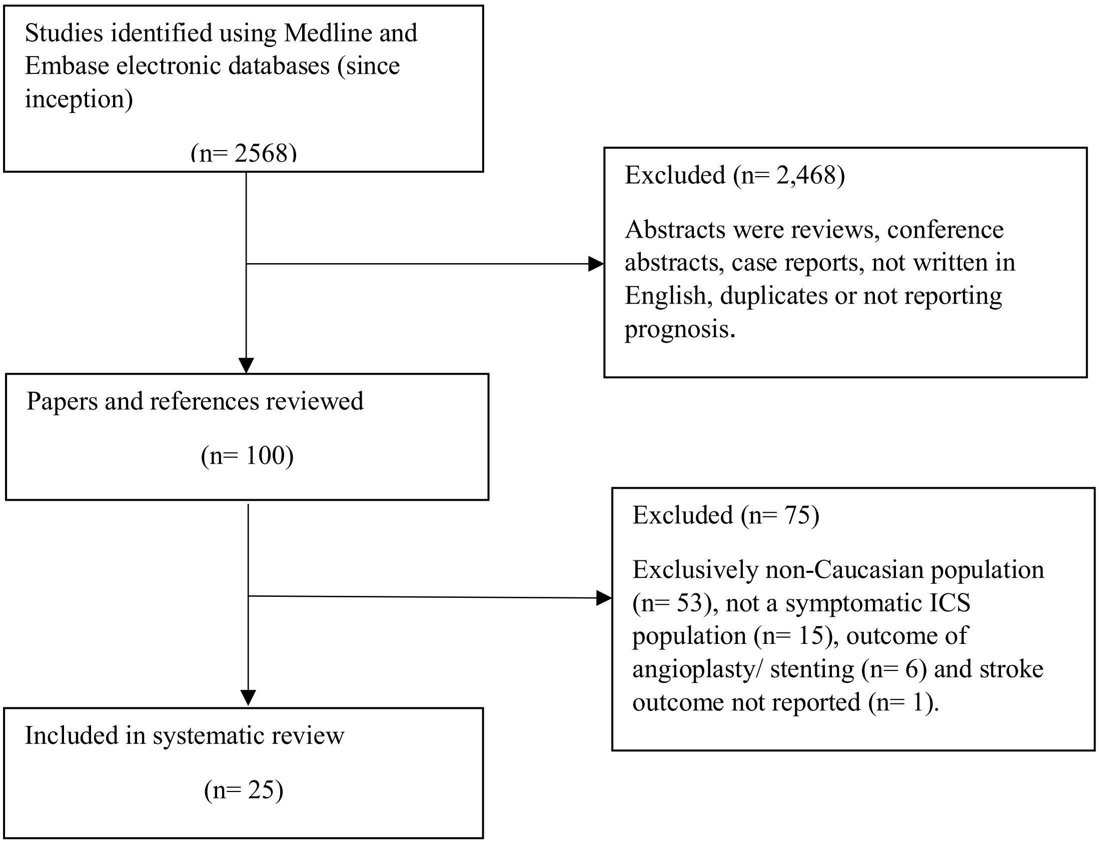

Medline

1. Intracranial arteriosclerosis/
2. (intracranial adj3 stenos*).ti,ab.
3. (intra-cranial adj3 stenos*).ti,ab.
4. (intracranial adj3 arteriosclero*).ti,ab.
5. (intracranial adj3 atherosclero*).ti,ab.
6. ((brain or cerebr*) adj3 atherosclero*).ti,ab.
7. ((brain or cerebr*) adj3 arteriosclero*).ti,ab.
8. 1 or 2 or 3 or 4 or 5 or 6 or 7
9. Follow up studies.sh.
10. Prognos*.tw.
11. Exp treatment outcome/
12. 9 or 10 or 11
13. 8 and 12

Embase

1. *brain atherosclerosis/
2. (intracranial adj3 stenos*).ti,ab.
3. (intra-cranial adj3 stenos*).ti,ab.
4. brain atherosclerosis/
5. (intracranial adj3 arteriosclero*).ti,ab.
6. (intracranial adj3 atherosclero*).ti,ab.
7. ((brain or cerebr*) adj3 atherosclero*).ti,ab.
8. ((brain or cerebr*) adj3 arteriosclero*).ti,ab.
9. 1 or 2 or 3 or 4 or 5 or 6 or 7 or 8
10. Exp *treatment outcome/
11. Outcome*.ti,ab.
12. Follow-up.mp.
13. Survival.tw.
14. Prognos*.tw.
15. 10 or 11 or 12 or 13 or 14
16. 9 and 15
17. Conference*.pt.
18. 16 not 17

Supplementary Figure 2*:* Kaplan-Meier curves comparing the risk of any recurrent ischaemic stroke in patients as reported in some previous studies with the risk of same-territory recurrent ischaemic stroke with SAMMPRIS inclusion criteria in patients with **a)** 50-99% symptomatic intracranial stenosis (ICS) and **b)** 70-99% symptomatic ICS.

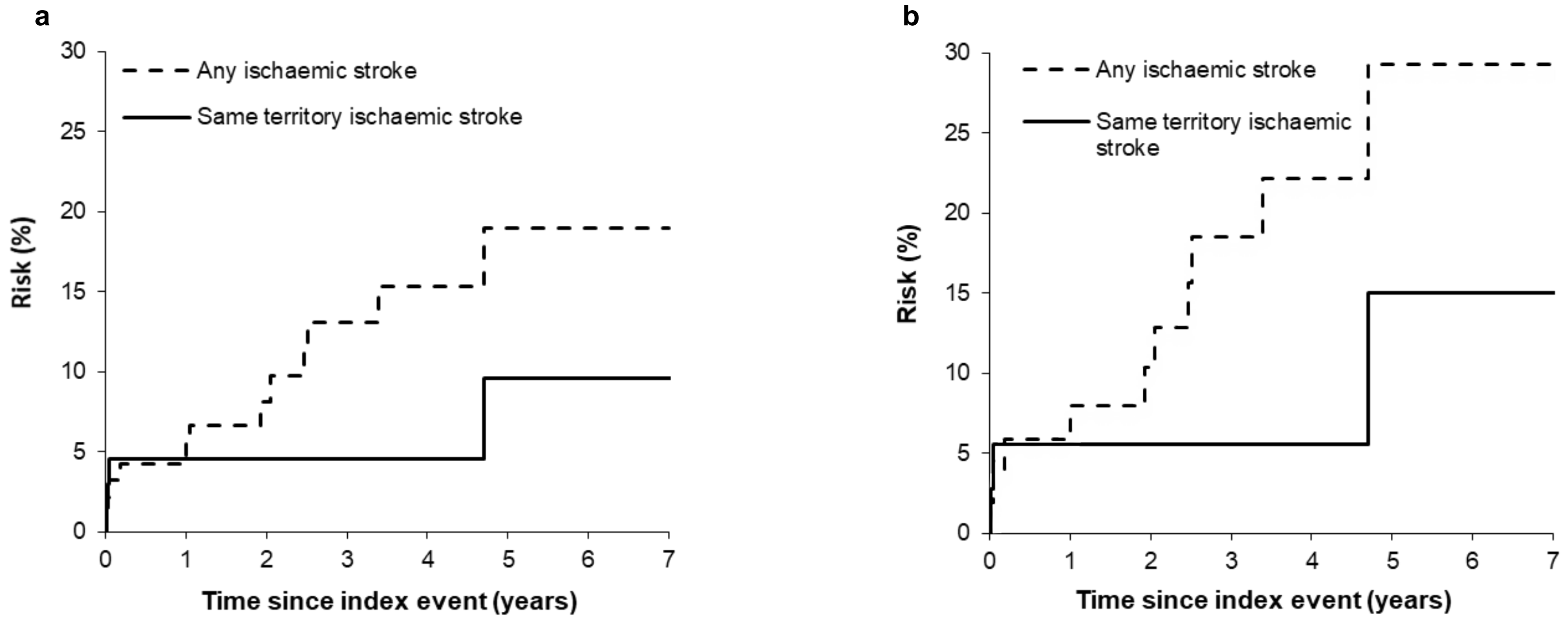

Any IS	94	80	59	43	35	20	10	3
Same territory IS	66	56	41	32	26	16	4	3

Any IS	52	45	35	26	19	9	5	2
Same territory IS	36	32	26	22	17	8	4	2

Supplementary References

1 Sacco RL, Kargman DE, Gu Q, Zamanillo MC. Race-ethnicity and determinants of intracranial atherosclerotic cerebral infarction. The Northern Manhattan Stroke Study. *Stroke* 1995; **26**: 14–20.

2 Wityk RJ, Lehman D, Klag M, Coresh J, Ahn H, Litt B. Race and sex differences in the distribution of cerebral atherosclerosis. *Stroke* 1996; **27**: 1974–80.

3 Thijs VN, Albers GW. Symptomatic intracranial atherosclerosis: outcome of patients who fail antithrombotic therapy. *Neurology* 2000; **55**: 490–7.

4 Weimar C, Goertler M, Harms L, Diener H-C. Distribution and outcome of symptomatic stenoses and occlusions in patients with acute cerebral ischemia. *Arch Neurol* 2006; **63**: 1287–91.

5 Meseguer E, Lavallee PC, Mazighi M, *et al.* Yield of systematic transcranial Doppler in patients with transient ischemic attack. *Ann Neurol* 2010; **68**: 9–17.

6 Weber R, Kraywinkel K, Diener H-C, Weimar C. Symptomatic intracranial atherosclerotic stenoses: prevalence and prognosis in patients with acute cerebral ischemia. *Cerebrovasc Dis* 2010; **30**: 188–93.

7 Lau AY, Wong KL, Lev M, Furie K, Smith W, Kim AS. Burden of intracranial steno-occlusive lesions on initial computed tomography angiography predicts poor outcome in patients with acute stroke. *Stroke* 2013; **44**: 1310–6.

8 Holzer K, Sadikovic S, Esposito L, *et al.* Transcranial Doppler ultrasonography predicts cardiovascular events after TIA. *BMC Med Imaging* 2009; **9**: 13.

9 Homburg PJ, Plas GJJ, Rozie S, van der Lugt A, Dippel DWJ. Prevalence and calcification of intracranial arterial stenotic lesions as assessed with multidetector computed tomography angiography. *Stroke* 2011; **42**: 1244–50.

10 von Weitzel-Mudersbach P, Paaske Johnsen S, Andersen G. Intra- and extracranial stenoses in TIA - findings from the Aarhus TIA-study: a prospective population-based study. *Persecptives Med* 2012; **1**: 207–10.

11 Amarenco P, Labreuche J, Lavallee PC. Patients with transient ischemic attack with ABCD2 <4 can have similar 90-day stroke risk as patients with transient ischemic attack with ABCD2 >/=4. *Stroke* 2012; **43**: 863–5.

12 Ovesen C, Abild A, Christensen AF, *et al.* Prevalence and long-term clinical significance of intracranial atherosclerosis after ischaemic stroke or transient ischaemic attack: a cohort study. *BMJ Open* 2013; **3**: e003724.

13 Ssi-Yan-Kai G, Nasr N, Faury A, *et al.* Intracranial artery stenosis or occlusion predicts ischemic recurrence after transient ischemic attack. *AJNR Am J Neuroradiol* 2013; **34**: 185–90.

14 Wolff V, Armspach J-P, Beaujeux R, *et al.* High frequency of intracranial arterial stenosis and cannabis use in ischaemic stroke in the young. *Cerebrovasc Dis* 2014; **37**: 438–43.

15 Logallo N, Naess H, Waje-Andreassen U, Thomassen L. Prevalence of intracranial stenosis in a Norwegian ischemic stroke population. *J Stroke Cerebrovasc Dis* 2014; **23**: 1611–5.

16 Tsivgoulis G, Vadikolias K, Heliopoulos I, *et al.* Prevalence of symptomatic intracranial atherosclerosis in Caucasians: a prospective, multicenter, transcranial Doppler study. *J Neuroimaging* 2014; **24**: 11–7.

17 Gouveia A, Sargento-Freitas J, Penetra J, *et al.* Recurrence in intracranial atherosclerotic disease: a stenosis-based analysis. *J Stroke Cerebrovasc Dis* 2014; **23**: 2080–4.

18 Baracchini C, Anzola GP, Cenciarelli S, *et al.* Italian symptomatic intracranial atherosclerosis study (ISIDE) : A multicenter transcranial ultrasound evaluation. *Neurol Sci Off J Ital Neurol Soc Ital Soc Clin Neurophysiol* 2016; **37**: 1645–51.

19 Hoshino T, Sissani L, Labreuche J, *et al.* Prevalence of Systemic Atherosclerosis Burdens and Overlapping Stroke Etiologies and Their Associations With Long-term Vascular Prognosis in Stroke With Intracranial Atherosclerotic Disease. *JAMA Neurol* 2018; **75**: 203–11.

20 Uchiyama S, Hoshino T, Sissani L, *et al.* Japanese Versus Non-Japanese Patients with Transient Ischemic Attack or Minor Stroke: Subanalysis of TIA registry.org. *J Stroke Cerebrovasc Dis* 2019; **28**: 2232–41.

21 Kappelle LJ, Eliasziw M, Fox AJ, Sharpe BL, Barnett HJ. Importance of intracranial atherosclerotic disease in patients with symptomatic stenosis of the internal carotid artery. The North American Symptomatic Carotid Endarterectomy Trail. *Stroke* 1999; **30**: 282–6.

22 von Sarnowski B, Schminke U, Tatlisumak T, *et al.* Prevalence of stenoses and occlusions of brain-supplying arteries in young stroke patients. *Neurology* 2013; **80**: 1287–94.

23 Mattioni A, Cenciarelli S, Biessels G, van Seeters T, Algra A, Ricci S. Prevalence of intracranial large artery stenosis and occlusion in patients with acute ischaemic stroke or TIA. *Neurol Sci Off J Ital Neurol Soc Ital Soc Clin Neurophysiol* 2014; **35**: 349–55.

24 Marquardt L, Kuker W, Chandratheva A, Geraghty O, Rothwell PM. Incidence and prognosis of > or = 50% symptomatic vertebral or basilar artery stenosis: prospective population-based study. *Brain* 2009; **132**: 982–8.

25 Klein IF, Lavallee PC, Mazighi M, Schouman-Claeys E, Labreuche J, Amarenco P. Basilar artery atherosclerotic plaques in paramedian and lacunar pontine infarctions: a high-resolution MRI study. *Stroke* 2010; **41**: 1405–9.

26 Chimowitz MI, Lynn MJ, Howlett-Smith H, *et al.* Comparison of warfarin and aspirin for symptomatic intracranial arterial stenosis. *N Engl J Med* 2005; **352**: 1305–16.

27 Mazighi M, Tanasescu R, Ducrocq X, *et al.* Prospective study of symptomatic atherothrombotic intracranial stenoses: the GESICA study. *Neurology* 2006; **66**: 1187–91.

28 Samaniego EA, Hetzel S, Thirunarayanan S, Aagaard-Kienitz B, Turk AS, Levine R. Outcome of symptomatic intracranial atherosclerotic disease. *Stroke* 2009; **40**: 2983–7.

29 Kozak O, Tariq N, Suri MFK, Taylor RA, Qureshi AI. High risk of recurrent ischemic events among patients with deferred intracranial angioplasty and stent placement for symptomatic intracranial atherosclerosis. *Neurosurgery* 2011; **69**: 333–4.

30 Nahab F, Kingston C, Frankel MR, *et al.* Early aggressive medical management for patients with symptomatic intracranial stenosis. *J Stroke Cerebrovasc Dis* 2013; **22**: 87–91.

31 Derdeyn CP, Chimowitz MI, Lynn MJ, *et al.* Aggressive medical treatment with or without stenting in high-risk patients with intracranial artery stenosis (SAMMPRIS): the final results of a randomised trial. *Lancet* 2014; **383**: 333–41.

32 Zaidat OO, Fitzsimmons B-F, Woodward BK, *et al.* Effect of a balloon-expandable intracranial stent vs medical therapy on risk of stroke in patients with symptomatic intracranial stenosis: the VISSIT randomized clinical trial. *JAMA* 2015; **313**: 1240–8.

33 Caliandro P, Reale G, Demchuk AM, *et al.* Symptomatic intracranial atherosclerotic disease: an ultrasound 2-year follow-up pilot study. *Neurol Sci Off J Ital Neurol Soc Ital Soc Clin Neurophysiol* 2018; **39**: 1955–9.

34 Hinton RC, Mohr JP, Ackerman RH, Adair LB, Fisher CM. Symptomatic middle cerebral artery stenosis. *Ann Neurol* 1979; **5**: 152–7.

35 Failure of extracranial-intracranial arterial bypass to reduce the risk of ischemic stroke. Results of an international randomized trial. *N Engl J Med* 1985; **313**: 1191–200.

36 Wechsler LR, Kistler JP, Davis KR, Kaminski MJ. The prognosis of carotid siphon stenosis. *Stroke* 1986; **17**: 714–8.

37 Arenillas JF, Molina CA, Montaner J, Abilleira S, Gonzalez-Sanchez MA, Alvarez-Sabin J. Progression and clinical recurrence of symptomatic middle cerebral artery stenosis: a long-term follow-up transcranial Doppler ultrasound study. *Stroke* 2001; **32**: 2898–904.

38 Kern R, Steinke W, Daffertshofer M, Prager R, Hennerici M. Stroke recurrences in patients with symptomatic vs asymptomatic middle cerebral artery disease. *Neurology* 2005; **65**: 859–64.

39 Moufarrij NA, Little JR, Furlan AJ, Leatherman JR, Williams GW. Basilar and distal vertebral artery stenosis: long-term follow-up. *Stroke* 1986; **17**: 938–42.

40 Pessin MS, Gorelick PB, Kwan ES, Caplan LR. Basilar artery stenosis: middle and distal segments. *Neurology* 1987; **37**: 1742–6.

41 Pessin MS, Kwan ES, DeWitt LD, Hedges TR 3rd, Gale D, Caplan LR. Posterior cerebral artery stenosis. *Ann Neurol* 1987; **21**: 85–9.

42 Qureshi AI, Ziai WC, Yahia AM, *et al.* Stroke-free survival and its determinants in patients with symptomatic vertebrobasilar stenosis: a multicenter study. *Neurosurgery* 2003; **52**: 1033–40.

43 Markus HS, Larsson SC, Kuker W, *et al.* Stenting for symptomatic vertebral artery stenosis: The Vertebral Artery Ischaemia Stenting Trial. *Neurology* 2017; **89**: 1229–36.
